# Household production and consumption impacts of foot and mouth disease at the Uganda-Tanzania border

**DOI:** 10.3389/fvets.2023.1156458

**Published:** 2023-06-05

**Authors:** Susan Diana Kerfua, Ashley Flynn Railey, Thomas Lloyd Marsh

**Affiliations:** ^1^National Livestock Resources Research Institute, National Agricultural Research Organisation, Entebbe, Uganda; ^2^Department of Sociology, Oklahoma State University, Stillwater, OK, United States; ^3^School of Economic Sciences and Paul G. Allen School for Global Health, Washington State University, Pullman, WA, United States

**Keywords:** foot-and-mouth disease, household survey, transboundary, consumption, production, Uganda, Tanzania

## Abstract

**Introduction:**

Foot-and–mouth disease (FMD) is a highly contagious viral disease that is endemic in East Africa. FMD virus infection incurs significant control costs and reduces animal productivity through weight loss, lowered milk yield, and potentially death but how household’s respond to these losses may differentially affect household income and food consumption.

**Methodology:**

To address this, we use unique data from a FMD outbreak to assess how household production and consumption activities change from before to during the outbreak. Data came from a 2018 survey of 254 households in selected Tanzanian wards and sub-counties in Uganda. The data includes household recall of before and during an outbreak in the past year on livestock and livestock product sales, milk and beef consumption, as well as related changes in market prices. We apply both difference-in-difference and change in difference ordinary least squares regressions with fixed effects to evaluate the impact of FMD on household production and consumption.

**Results and discussion:**

We find that households reported the largest reductions in livestock and livestock product sales, followed by reduced milk consumption and animal market prices. The changes in household income from livestock sales appears to be driven by FMD virus infection within the household herd while changes in market prices of substitute protein sources are primary associated with changes in milk and beef consumption. The role of widespread market price effects across both infected and uninfected herds and countries, tends to suggest that stabilizing prices will likely have a large impact on household nutritional security and income generation. We also propose that promoting diversity in market activity may mitigate differing impacts on households in FMD endemic regions.

## Introduction

Over 70% of the population in Tanzania and Uganda is employed in the livestock industry (1, 2). The pervasiveness of livestock in East Africa provides opportunities to enhance individual household livelihoods while progressing the general development of the region. Households receive economic benefits from livestock in the form of insurance (3–5) and as a source of income generation (6, 7). Livestock can also provide nutritional benefits through the supply of animal-based proteins (8–11) or increase access to diverse foods and resources through informal networks (12, 13). Yet, the constant threat of livestock diseases across Africa undermines the full realization of these benefits. Indeed, transboundary, highly contagious diseases are considered a significant barrier to the growth of the livestock sector in Uganda and Tanzania (14) and poverty reduction broadly among livestock owning households (15).

Foot-and-mouth disease (FMD) represents one of the most economically damaging infectious transboundary diseases in livestock that continues to threaten livelihoods in East Africa (16). The rapid spread of the virus across high-value livestock holdings, such as cattle, sheep, and goats, is accompanied by widespread disease control costs, reduced livestock production, and denied trade opportunities (17). Estimates suggest that the largest production losses due to FMD occur in Africa, at around $830 million or 17% of the total, worldwide annual costs (18). Production losses hinder sector growth regionally but livestock owning households incur the most immediate impacts through increased control costs and lost income (19–21). Importantly, FMD is endemic in East Africa (22, 23) such that households have previous experience with the disease to identify an outbreak but also lack sufficient tools to fully prevent infection or protect against production losses. Frequent inter-herd interactions coupled with limited availability of vaccines constrain East African households from preventing endemic diseases (24), such as FMD. While households expect an outbreak, the exact timing of the outbreak and magnitude of the impact prevents households from changing practices in anticipation of the outbreak (25). Thus, defining how FMD affects households in areas with large livestock populations is important to improving our estimates on the burden of disease, as well as strengthen our understanding of how to reduce household vulnerability to livestock disease.

To this end, our study leverages a unique dataset of livestock owning households from before and during an FMD outbreak at the Uganda-Tanzania border. We assessed FMD effects on indicators of household economic benefits through livestock and livestock product sales, along with evaluating household consumption through changes in intake of milk, beef, and related products. With data from two time periods across both households reporting FMD virus infections (treatment) and no infections (control), we employ difference-in-difference and change in difference estimations to evaluate the relationship between FMD and changes in household and market behaviors. Our analysis builds on existing knowledge of FMD impacts in endemic regions by contributing evidence on disease impacts over time and emphasizing the dual role of household livestock ownership for production and consumption (26). Our analysis intends to improve household benefits from livestock by providing evidence to better design policies and interventions for livestock disease prevention and control.

## Materials and methods

### Study design

**Figure 1:**
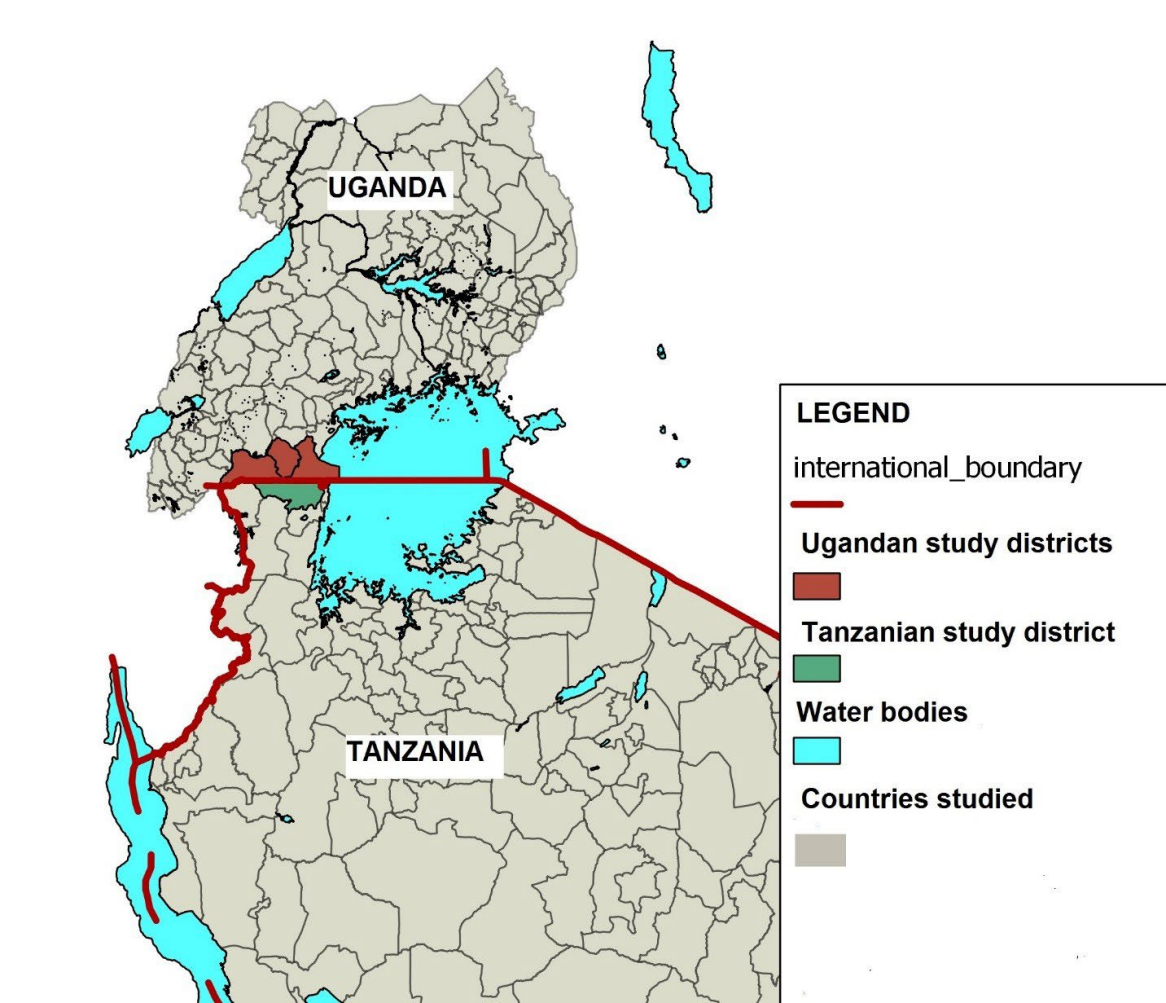
Map of Uganda and Tanzania showing the districts in Uganda (in red) and that in Tanzania (in green) where the study was conducted.

The study was conducted in Kyaka and Nsunga wards of Missenyi district in Tanzania and in the sub-counties of Endinzi in Isingiro district, Lwamaggwa in Rakai district, and Kakuuto in Kyotera district in 2018 (Figure 1).

A total of 288 samples were estimated for inclusion in the study. Sample size calculation was based on the formula by Taherdoost (27) that required at least 84 households per district, however our study considered interviewing 96 HHs per district. The study assumed an impact percentage of 33% for settings in which FMD was endemic (18). Households were randomly selected from within sub-counties and wards where an FMD outbreak had previously been verified (28) and based on a list of livestock keepers compiled and provided by the District Veterinary Officers. Sampling occurred in a two-stage process by first selecting clusters, then households, with Uganda more intensively sampled to facilitate analysis. Households provided retrospective accounts on household and market behavior before and after the FMD outbreak that had occurred in the past year (July–August 2017). Out of 288 HHs, 264 households were sampled (170 households from Uganda and 85 households from Tanzania), 256 completed the questions for the analysis (97% response rate). The households that reported not knowing whether there was an FMD outbreak (*n* = 2) were excluded from the analysis for a sample size of 254. Data collected locally at the markets and through discussions with local leaders helped reduce recall bias on market data. Additional missing data appeared seemingly at random, with missing variables occurring in no more than 10% of the variables included in the analysis. Data was collected using a smart phone application called Kobo Collect, which is an OpenDataKit system and saved accordingly. Analysis of data occurred in R. The authors confirm that the ethical policies of the journals’ author guidelines page have been adhered to and permission to conduct the study was granted by the Tanzania Commission for Science and Technology (Permit No: 2016-277-NA-2016-214).

### Data

The primary focus of our study was to evaluate the effects of FMD on household production and consumption of livestock products. Household revenue from livestock and livestock product sales capture households’ main wealth and income generation from market activities. Livestock sales and livestock product sales were reported per month. Livestock products included milk, animal hides, ghee, and manure. We assessed changes in quantity and content of household consumption through changes in household consumption of milk, measured per *tumpeco* per day, or a traditional cup measurement in Uganda and parts of Tanzania (about 1/2 liter), and beef consumption (per kilo) consumed per week.

Whether the herd was infected with FMD in the last year was used to proxy FMD occurrence in the herd (1 = FMD virus infection; 0 = no FMD virus infection). Additional data on the changes in market prices captured market-based effects that can accompany widespread disease outbreaks and changes in the availability of food products. Specifically, households reported on prices for milk per serving of milk (about ½L), beef per kilogram, per chicken, per egg, and per kilogram of beans. Input costs for FMD cattle vaccines and therapeutic antibiotic treatments during an outbreak were included in the sales models. Finally, data on the number of adults living in the household, the proportion of under 5 years old in the household, and the number of cattle, sheep, and goats kept at the household control for the diminishing returns of additional livestock holdings *per capita*. We also control for whether livestock keeping is the household’s primary source of income (1 = yes; 0 = no).

Data transformations occurred to translate livestock sales data and market prices into Ugandan shillings (UgX). We adjusted values from Tanzanian households by 1.6 to reflect the exchange rate in 2018. Missing price data was imputed based on reported average prices in the district. For the change in difference models, we took the difference in the values from before (time = 0) to during the outbreak (time = 1). Households that reported not selling or consuming the product across both time-periods were included as zeros and were treated as equivalent to households that reported no difference from before to during an outbreak.

### Analytical strategy

We combined summary statistics and regression analyzes to examine household income and food consumption in selected districts located on the Uganda-Tanzania border before and during an FMD outbreak. We first generated summary statistics of household characteristics by country, followed by descriptive statistics of the reported changes due to an FMD outbreak in household livestock activities and market prices. Fisher’s exact or Pearson’s chi-squared tests were used to determine the strength of the association between the comparison groups in both cases. For continuous variables, we used two-sided t-tests to compare means across the different groups.

Our main analyzes evaluated the difference from before and during the FMD outbreak across household and market factors using (1) standard difference-in-difference (29) and (2) the change in differences approaches (30). Both analyzes are ordinary least squares regression. The effects of FMD were evaluated through two measures of household income and two measures of household consumption: livestock sales in the past year, livestock product sales in the past year, milk consumption per day, and beef consumption per week. The difference-in-difference models captured the average effect of FMD virus infection on the households that reported FMD, representing those in the treatment group compared to those in the control, or no FMD virus infection group. The endemic nature of FMD and the limited availability of preventative measures in the region facilitates the comparison through the common trends assumption by suggesting that neither group of households can effectively prevent FMD (group invariant) nor that the groups tend to drastically change their livestock management practices during an outbreak (time invariant). The change in difference models then allowed us to define potential dynamic changes in the market and household inputs that were related to the FMD outbreak. For both approaches, we employed country fixed effects to account for remaining unobserved country-level effects.

The model specifications reflect the best fit based on comparing separate and pooled models as well as sample strategy. The additional model specifications are in the Supplementary material. Results from the analyzes are reported with marginal effects whereby the continuous variables reflect the elasticities at the mean of the variable and binary, categorical variables are percentage changes going from one level to the next.

## Results

### Summary statistics

Table 1 provides the summary statistics of the households in the sample. At the time of the study, 67% (*n* = 170) of the households had experienced an FMD outbreak on their farm. Households reported an average herd size of 54 cattle, sheep, and goats but with significant variation, ranging from currently not owning animals to owning 71 animals. The average household size was 8.7 persons which included an average of 2 children below 5 years old. Households across the two countries and primary source of household income differed by FMD herd occurrence (*p* value <0.01 and 0.03, respectively).

**Table 1:** Household summary statistics (*n* = 254).

	Overall *n* = 254	FMD		Value of *p*^‡^
		No *n* = 83^†^	Yes *n* = 170^†^	
Tanzania	83 (33%)	39 (47%)	45 (26%)	<0.01
Uganda	169 (67%)	44 (53%)	125 (74%)	
Household size, m(sd)	8.7 (6.7)	8.7 (6.5)	8.7 (6.8)	0.80
Proportion of household <5-yrs old, m(sd)	1.6 (2.1)	0.2 (0.2)	0.2 (0.1)	0.60
Herd size (cattle, sheep, goats), m(sd)	54 (76)	47 (67)	57 (81)	0.32
Primary income-livestock	84 (33%)	20 (24%)	64 (38%)	0.03

^†^*n*/*N* (%); Mean (sd). ^‡^Pearson’s Chi-squared test or Fisher’s exact test for categorical variables and two-sided *t*-tests for continuous data.

Table 2 shows the household reported changes in production and consumption activities from before to during the FMD outbreak. Overall households reported a decrease in sales and prices, except for beef consumption per week (2.9 vs. 3.0 kilo/week before/during; *p* value = 0.11), chicken consumption (1 time per day vs. 0.8 time before/after; *p* value 0.20), and the price per FMD vaccination (1950 UgX vs. 2,460 UgX per animal before/during *p* value <0.01). Of the reported changes, livestock and livestock product revenues saw the greatest decrease (63 and 70%, respectively; *p* value <0.01 for both). The next greatest changes were reported for milk consumption (49% decrease), the price of bulls, cows, sheep, and goats (41–48% decrease), the price of beef (38% decrease), and the price of chicken (39% decrease) (*p* value <0.01 for all changes).

**Table 2:** Descriptive statistics of household livestock activities and market prices from before to during an FMD outbreak, *n* = 254.

	Before, *n* = 254^†^	During, *n* = 254^†^	Difference	Value of *p*^‡^	% Change^§^
Livestock Sales Only (UgX)	940,000 (1000000)	350,000 (500000)	−590,000	<0.01	63%
Livestock and Livestock Product Sales (UgX)	530,000 (870000)	160,000 (260000)	−370,000	<0.01	70%
Milk Consumption (servings/day)	7.5 (9.4)	3.8 (5.4)	−3.7	<0.01	49%
Beef Consumption (kilo/week)	2.9 (3.7)	3.0 (5.6)	0.1	0.11	3%
Chicken Consumption (#/day)	1.0 (1.4)	0.8 (1.3)	−0.2	0.20	20%
FMD vaccine cost (per animal)	1950 (1178)	2,460 (1317)	510	<0.01	26%
Milk Price (serving)	590 (150)	530 (250)	−60	<0.01	10%
Beef Price (kilo)	8,800 (1500)	5,500 (2000)	−3,300	<0.01	38%
Bean Price (kilo)	2000 (520)	1900 (890)	−100	0.01	5%
Egg Price (kilo)	410 (100)	330 (150)	−80	<0.01	20%
Chicken Price (per animal)	23,000 (5400)	14,000 (4,600)	−9,000	<0.01	39%
Bull Price (per animal)	1,500,000 (790000)	820,000 (450000)	−680,000	<0.01	45%
Cow Price (per animal)	1,200,000 (490000)	710,000 (370000)	−490,000	<0.01	41%
Goat Price (per animal)	110,000 (46000)	57,000 (29000)	−53,000	<0.01	48%
Sheep Price (per animal)	150,000 (52000)	87,000 (41000)	−63,000	<0.01	42%

^†^Mean (SD) ^‡^Chi-square or exact test or two-sided *t*-test, where appropriate. ^§^Percent change from before FMD outbreak. All prices in UgX = Ugandan shillings (US $ 1 = UgX 3,600; Tz Shilling 0.6 = UgX 1); kilo = kilogram; # = number; milk serving = 1 glass, or about ½L.

### Foot-and–mouth disease impacts on household income

The results for the impact of FMD virus infection on livestock production appear in Table 3. FMD was estimated to reduce livestock sales by 250,000 UgX (*p* value = 0.09) in those households who experienced FMD within the herd during the outbreak (treatment effect). To calculate the average livestock sales for the households that experienced FMD in the herd during the outbreak, we added the intercept and FMD coefficient to the change over time due to the treatment (FMD x Time) and in the households not reporting FMD (Time). The result was a loss in sales (−112,000 UgX), which would imply zero income or debts. In contrast, evaluating the counterfactual whereby FMD in the household does not affect livestock sales, we would expect the average livestock sales income of households reporting FMD to be 98,000 UgX during the outbreak.

**Table 3:** Difference-in-difference estimation of FMD on household livestock sales income (*n* = 254).

	Livestock sales				Livestock product sales			
	Estimate	95% CI		*P* value	Estimate	95% CI		*P* value
Intercept	398,000	215,000	581,000	<0.01	230,000	76,300	383,000	<0.01
FMD	198,000	−4,190	401,000	0.07	160,000	−11,000	332,000	0.07
Time	−428,000	−660,000	−196,000	<0.01	−244,000	−440,000	−47,600	0.02
FMD x Time	−250,000	−530,000	37,900	0.09	−190,000	−431,000	50,300	0.12
Country FE	Yes				Yes			
*R* ^2^	0.52				0.32			

Ordinary least squares regression with country fixed effects. Coefficient estimates interpreted as discrete changes from reference category. Reference categories: No reported FMD in household; Tanzania; Time before outbreak. CI = Confidence Interval.

The effects of FMD through changes in market and household control decisions appear in Table 4. A change in the market price per bull was associated with an increase in livestock sales by 0.35 UgX during a FMD outbreak (*p* < 0.01). In contrast, a change in the price of a chicken was associated with a decrease in livestock sales of 19.0 UgX (*p* = 0.07). Sales of livestock products were positively related to a change in beef prices (50.5 UgX, *p* = 0.08) and egg prices (2,030 UgX, *p* < 0.01). Compared to households that reported engaging in multiple agricultural practices beyond livestock, relying on livestock as a primary income source was associated with a − 214,000 UgX in livestock product sales (*p* = 0.09). An increase in therapeutic antibiotic treatment costs during the outbreak were related to increased sales revenue from livestock products (*p* = 0.03).

**Table 4:** Relationship between change in market prices and household livestock sales income after an FMD outbreak (*n* = 254).

	Livestock sales				Livestock product sales			
	Estimate	95% CI		Value of *p*	Estimate	95% CI		Value of *p*
FMD								
Yes	34,200	−187,00	255,000	0.80	−182,000	−428,000	64,400	0.15
Herd size	15,700	−28,800	60,200	0.50	30,000	−18,900	79,600	0.20
Family size	72,100	−103,000	247,000	0.40	−20,600	−214,000	173,000	0.80
Proportion < 5	−9,201	−147,000	128,000	0.90	−70,500	−226,000	85,400	0.40
FMD vaccine cost	44.7	−44.7	134	0.30	−21.3	−121	78.7	0.70
Antibiotic treatment costs	0.38	−5.62	6.38	0.90	7.67	0.89	14.5	0.03
Primary income-livestock								
Yes	−38,000	−257,000	181,000	0.70	−214,000	−463,000	34,700	0.09
Bull price (per animal)	0.35	0.17	0.54	<0.01	0.14	−0.074	0.35	0.20
Beef price (per kilo)	−18.4	−67.7	30.9	0.50	50.5	−5.14	106	0.08
Chicken price (per animal)	−19.0	−39.8	1.84	0.07	−18.7	−42.1	4.70	0.12
Egg price (per egg)	336	−838	1,510	0.60	2,030	647	3,410	<0.01
Bean price (per kilo)	23.9	−112	160	0.70	−21.2	−172	130	0.80
Milk price (per serving)	355	−185	895	0.20	441	−171	1,050	0.20
Country fixed effects	Yes				Yes			
*R* ^2^	0.46				0.32			

Ordinary least squares regression with country fixed effects. Reference categories: No reported FMD in household; no formal head of household; other income beyond livestock. Household and herd size are exponentiated. All prices in UgX = Ugandan shillings (US $ 1 = UgX 3,600). CI = Confidence Interval.

### Foot-and–mouth disease impacts on household food consumption

The results for the impact of FMD virus infection on household consumption appear in Table 5. The difference-in-difference effect was not statistically significant in the milk and meat models, suggesting no difference between the treatment (FMD occurrence in the herd) and control (no virus infection) group. Unlike the household income models, FMD occurrence in the herd was associated with lower milk consumption compared to households without FMD (2.22 servings per day; *p* value = 0.02). Before the outbreak, this translated into households with FMD consuming 3.11 servings per day compared to 5.33 in the households not reporting FMD. During the outbreak, households not reporting FMD saw a reduction in milk consumption by −3.75 servings (*p* value <0.01) for an average daily consumption of 1.58 servings or 30% of their pre-FMD outbreak consumption levels. Beef consumption widely varied both before and after the FMD outbreak.

**Table 5:** Difference in difference estimation of FMD on household consumption (*n* = 254).

	Milk consumption				Beef consumption			
	Estimate	95% CI		*P* value	Estimate	95% CI		*P* value
Intercept	5.33	3.60	7.06	<0.01	4.88	3.80	5.97	<0.01
FMD	−2.22	−4.15	−0.29	0.02	0.01	−1.21	1.22	>0.90
Time	−3.75	−5.96	−1.53	< 0.01	0.43	−0.96	1.83	0.54
FMD x Time	0.04	−2.67	2.74	0.98	−0.59	−2.30	1.11	0.50
Country FE	Yes				Yes			
R2	0.45				0.34			

Ordinary least squares regression with country fixed effects. Coefficient estimates interpreted as discrete changes from reference category. Reference categories: No reported FMD in household; Tanzania; Time before outbreak. CI = Confidence Interval.

The effects of changes in market and household control decisions on household consumption appear in Table 6. Given this is an agricultural household production situation, then we have no *a priori* expectations on the signs of the reported parameters. For milk consumption, a change in beef prices was associated with the largest change in milk consumption (0.001 servings; *p* value = 0.02), followed by a change in the price of chickens (−0.0002 servings; *p* value = 0.02) and livestock sales income (<0.0001 serving; *p* value <0.01). For perspective, households reported that beef prices changed on average by 3,300 UgX/kilo from before to during an outbreak. At this price, we would expect a change of 3.3 servings of milk per day. In our beef consumption model, the change in price per chicken was similarly inversely related to the change in beef consumption (−0.0002 kilo; *p* value = 0.03) while the change in the prices per egg and market bull were positively related to changes in beef consumption (0.01 kilos; *p* value = 0.01; <0.0001 kilo per UgX; *p* value = 0.02, respectively).

**Table 6:** Relationship between change in market prices and household consumption after an FMD outbreak.

	Milk consumption				Beef consumption			
	Estimate	95% CI		Value of *p*	Estimate	95% CI		Value of *p*
FMD								
Yes	−0.39	−2.18	1.40	0.70	−0.75	−2.17	0.66	0.30
Herd size	−0.14	−0.50	0.23	0.50	0.08	−0.21	0.37	0.60
Family size	0.51	−0.94	1.96	0.50	−0.69	−1.83	0.46	0.20
Proportion < 5	−0.44	−1.58	0.71	0.50	−0.67	−1.57	0.24	0.15
Primary income-livestock								
Yes	0.02	−1.80	1.84	>0.90	0.65	−0.79	2.10	0.40
Livestock sales	0.0	0.0	0.0	<0.01	0.0	0.0	0.0	0.90
Bull price (per animal)	0.0	0.0	0.0	>0.90	0.0	0.0	0.0	0.02
Beef price (per kilo)	0.001	0.0001	0.001	0.02	−0.0002	−0.001	0.0	0.20
Chicken price (per animal)	−0.0002	−0.0004	0.0	0.02	−0.0002	−0.0003	0.0	0.03
Egg price (per egg)	0.003	−0.007	0.013	0.50	0.01	0.003	0.02	0.01
Bean price (per kilo)	−0.001	−0.002	0.001	0.40	−0.001	−0.001	0.0003	0.20
Milk price (per serving)	−0.0004	−0.005	0.004	0.90	0.003	−0.001	0.01	0.13
Country Fixed Effects	Yes				Yes			
*R* ^2^	0.35				0.11			

Ordinary least squares regression with country fixed effects. Reference categories: No reported FMD in household; no formal head of household; other income beyond livestock. Household and herd size are exponentiated. All prices in UgX = Ugandan shillings (US $ 1 = UgX 3,600). CI = Confidence Interval.

## Discussion

Our study contributes to growing research on control of endemic, transboundary diseases by adding empirical evidence on disease impacts across households and local markets (31). Access to unique data on household and market activities from before and during FMD outbreaks in Uganda and Tanzania allowed us to identity FMD virus infection as influencing household production directly and household consumption indirectly. Specifically, we identified FMD virus infection as directly reducing household income from livestock sales by a magnitude of 30% compared to the counterfactual of no FMD occurrence in the herd, and having non-trivial, indirect effects on household consumption through changes in the prices of related and substitute products. These results support evidence to suggest that production losses due to disease can affect households with herds infected by the FMD virus and those not reporting infection (30), as well as have differential impacts within households (32, 33).

Overall, households reported decreases in revenue from livestock sales by 63% and a decrease in livestock product sales by 70%. These results resonate with a study by Rutagwenda (21) which showed that farmers in Kumi and Mbarara districts in Uganda had significant income losses during an FMD outbreak from reduced livestock sales. We find evidence to suggest that FMD virus infection directly reduces livestock sales but that additional changes in sales likely occur through changes in market prices, including for milk, beef, and bulls. This highlights the fact that the impact of FMD cuts across both households with virus infected and uninfected herds and can affect commodity prices for substitute and related goods (34). The decrease in livestock prices and sales in both countries could result from changes in supply of quality, healthy animals or from changes in demand for animals (35, 36). Recognizing these critical spillovers across markets and households highlights the need for disease reporting at the farmer and policy level and may help reduce the widespread distribution of disease impacts during an outbreak (37).

We further find evidence to support extant literature that suggests that FMD primarily affects milk consumption (38, 39). Households in Uganda and Tanzania reported reducing milk consumption by nearly half during an outbreak but maintaining pre-outbreak beef consumption levels. We find limited evidence of the effect of FMD virus infection on milk consumption through our difference-in-difference model. Instead, the relationship between changes in market prices for beef, chicken, and livestock sales and milk consumption would indicate that changes in milk consumption were driven by market shifts in the availability of livestock products. Households that reported FMD occurrence in their herds likely saw reduced milk production compared to households not reporting FMD occurrence as this is a primary effect of the FMD virus (40). This could be due to the endemic nature of the disease, or that that we do not delineate between consumers and producer/consumers (41). This may further be the byproduct of selling limited milk supplies to finance immediate needs within households that primarily sell milk compared to those that primarily consume or purchase milk (41). Milk cooperatives also have a strong presence in Uganda and ensure the market availability of milk but may simultaneously be affecting household milk consumption or the decision to sell milk, beef, animals, or consume the byproducts (42). The influence of market factors on milk consumption is further supported by knowing that milk was sold in the Ugandan districts regardless of the movement restrictions on livestock and other livestock products. Given that the price effects cut across both groups of households and countries, stabilizing prices will likely have a larger impact on household nutritional security than directing relief directly to infected households. Diversity in market activity may then help mitigate differing impacts on households in endemic regions. However, defining how milk is allocated within the households would help capture the trade offs that exist between income and consumption to better identify the magnitude of FMD effects to distribute control efforts. Especially as most households have at least two children below the age of five, ensuring the continued consumption of milk will have large nutritional impacts and align with preferences for milk over other protein sources.

The impacts of FMD on household livestock activities reflects the importance of devising transboundary control strategies in the region. Over 60% of households across Uganda and Tanzania reported an FMD outbreak supporting evidence to suggest that FMD virus infection is pervasive in East Africa (22, 43). The widespread persistence and impacts of FMD along with the high diversity of FMD serotypes in circulation between 2013 and 2018 (22) likely reflect the high volume of trade within our study regions (44). Coupled with increasing evidence on practical approaches for proactive vaccination for FMD in East Africa (45–47) and willingness to pay for approaches to improve vaccine matching (25), there is a strong need for transboundary collaborative efforts and policies to address the circulation of the FMD virus in East Africa.

Our analysis optimizes on existing laboratory confirmations of FMD in parts of Uganda and Tanzania but is limited by collecting retrospective data. Recall error, social desirability bias, or other social biases may influence the accuracy of the estimates. However, the persistence of FMD in the area and the resulting familiarity among the households of the disease and market response suggests our approach may broadly capture average effects in the absence of widespread and available records on fluctuations in markets and household consumption. Our analysis is additionally limited by sample size. Particularly regarding the impacts of FMD on beef consumption and across types of households based on income generation (i.e., livestock only, livestock and agriculture, or income levels). Further evaluations into variation across household types of consumers and/or producers may elucidate further market impacts. Similarly, next steps should assess cross-product effects to better understand the relative trade offs in market activity after an outbreak. As is, we contribute initial evidence on the impact of FMD on local markets, especially revealing the direct impacts on household milk consumption and potential for income from livestock sales.

## Conclusion

Our paper shows how livestock disease directly and indirectly impacts household production and consumption activities. Specifically, we found that FMD results in changes to local market prices, which then can indirectly affect household consumption patterns while FMD occurrence in the herd directly affects household livestock sales. Investments in livestock health, particularly through vaccination, represents a potential intervention to prevent disease outbreaks that have been shown to be feasible and accepted approaches in eastern Africa for FMD (45). Importantly, these interventions may further have positive impacts on human capital and economic growth (7) to improve the livelihoods of livestock owning households. In a broader sense, these results contribute to the growing knowledge of animal disease burden across the globe (48, 49).

## Data availability statement

The raw data supporting the conclusions of this article will be made available by the authors, without undue reservation.

## Ethics statement

The studies involving human participants were reviewed and approved by Tanzania Commission for Science and Technology (Permit No: 2016-277-NA-2016-214). The patients/participants provided their written informed consent to participate in this study.

## Funding

This work was funded by the Program for Enhancing Health and Productivity in Livestock under the Bill and Melinda Gates Foundation (Project code: 02021059–048–301-4001-P023-j01S01-C21).

## Author contributions

SK made substantial contributions to conception and design, acquisition of data, and analysis and interpretation of data, reviewing and editing the manuscript. AR was involved in drafting the manuscript, major analysis and interpretation of data and revising it critically for important intellectual content. TM was involved in conception and design, analysis and interpretation of data and critical revision of the manuscript for important intellectual content. All authors contributed to the article and approved the submitted version.

## Conflict of interest

The authors declare that the research was conducted in the absence of any commercial or financial relationships that could be construed as a potential conflict of interest.

## Publisher’s note

All claims expressed in this article are solely those of the authors and do not necessarily represent those of their affiliated organizations, or those of the publisher, the editors and the reviewers. Any product that may be evaluated in this article, or claim that may be made by its manufacturer, is not guaranteed or endorsed by the publisher.

## Supplementary material

The Supplementary material for this article can be found online at:

https://www.frontiersin.org/articles/10.3389/fvets.2023.1156458/full#supplementary-material
