# Supplementary material for: Household production and consumption impacts of foot and mouth disease at the Uganda-Tanzania border
Source: Front Vet Sci. 2023 Jun 5;10:1156458. doi: 10.3389/fvets.2023.1156458 (PMC10277485; doi:10.3389/fvets.2023.1156458)
Supplement: Supplementary file 1 [file Table_1.pdf]

Supplementary material

Household production and consumption impacts of FMD at the Uganda Tanzania border

Table S1 Relationship between change in market prices and livestock activities after an FMD outbreak

	Livestock Sales				Livestock Product Sales			
	Uganda		Tanzania		Uganda		Tanzania	
	Beta	p-value	Beta	p-value	Beta	p-value	Beta	p-value
FMD								
Yes	2,977	>0.9	90,349	0.7	-158,470	0.3	-259,342	0.3
Herd size	11,219	0.8	-1,794	>0.9	14,829	0.7	34,010	0.4
Family size	128,183	0.2	-56,329	0.7	48,382	0.7	-141,668	0.5
Proportion household <5 yrs old	-18,330	0.8	32,960	0.8	-41,555	0.7	-94,914	0.6
FMD vaccine cost	10	0.9	52.5	0.5	10.5	0.9	-0.0035	>0.9
Antibiotic treatment costs	0.79	0.8	-0.3726	>0.9	5.85	0.1	19.3	0.08
Primary Income-Livestock								
Yes	-56,465	0.6	161,516	0.6	-200,670	0.12	-360,271	0.4
Bull price (per animal)	0.38	0.001	0.37	0.046	0.18	0.2	0.030	0.9
Beef price (per kilo)	5.2	0.9	-91.6	0.11	50.0	0.1	44.6	0.5
Chicken price (per animal)	-22	0.09	-10.2	0.6	-16.3	0.2	-10.9	0.7
Egg price (per egg)	402	0.6	416	0.7	1,600.00	0.05	2651	0.07
Bean price (per kilo)	47	0.6	-16.3	>0.9	3.56	>0.9	-125	0.4
Milk price (per serving)	305	0.3	463	0.4	369	0.3	702	0.3
Country Fixed Effects	Yes		Yes		Yes		Yes	
No. Obs.	160		83		150		83	
R^2	0.13		0.15		0.16		0.17	

Notes: Reference categories: No reported FMD in household; no formal head of household; other income beyond livestock. Household and herd size are exponentiated. All prices in UgX=Ugandan shillings (US $ 1= UgX 3600). [1] CI = Confidence Interval

Table S2 Relationship between change in market prices and household consumption after an FMD outbreak with household controls

	Milk consumption				Beef consumption			
	Uganda		Tanzania		Uganda		Tanzania	
	Beta	p-value	Beta	p-value	Beta	p-value	Beta	p-value
FMD								
Yes	0.4753	0.7	-1.7515	0.2	-1.6819	0.051	0.4778	0.7
Herd size	-0.0645	0.9	-0.0601	0.8	0.0915	0.7	0.1735	0.5
Family size	0.3024	0.8	0.989	0.4	-0.8816	0.2	-0.502	0.7
Proportion household <5 yrs old	-0.7857	0.3	0.1595	0.9	-0.3531	0.5	-1.4283	0.14
Primary Income-Livestock								
Yes	0.5613	0.6	-3.2633	0.2	0.969	0.2	-1.2953	0.6
Livestock sales	0	0.034	0	0.033	0	>0.9	0	0.9
Bull price (per animal)	0	0.8	0	0.6	0	0.2	0	0.04
Beef price (per kilo)	0.0005	0.053	0.0006	0.2	-0.0002	0.4	-0.0002	0.6
Chicken price (per animal)	-0.0002	0.04	-0.0002	0.3	-0.0002	0.051	-0.0002	0.3
Egg price (per egg)	0.0043	0.5	0.0033	0.7	0.0092	0.038	0.0139	0.1
Bean price (per kilo)	-0.0005	0.5	-0.0003	0.7	-0.0005	0.3	-0.0005	0.5
Milk price (per serving)	0	>0.9	-0.0003	>0.9	0.0025	0.2	0.0024	0.5
Country Fixed Effects	Yes				Yes			
No. Obs.	160		83		160		83	
R^2	0.13		0.16		0.10		0.17	

Notes: Reference categories: No reported FMD in household; no formal head of household; other income beyond livestock. Household and herd size are exponentiated. All prices in UgX=Ugandan shillings (US $ 1= UgX 3600). [1] CI = Confidence Interval
